# Practical Notes and Formulæ

**Published:** 1886-06-20

**Authors:** 


### PRACTICAL NOTES AND FORMULÆ.

**Useful Formulæ.**—The following formulæ have been used by me for the past three years with uniformily successful results. In the chlorosis of young girls, in all forms of simple anæmia, in amenorrhœa due to anæmia, and in the nervous debility and neuralgias dependent on an anæmic condition, I have never seen a drug, or combination of drugs or chemicals, equal to those given below for rapidly increasing the number of red blood-globlues, and bringing the roses to the cheeks of the pale and chlorotic :

℞. Strychniæ sulphatis..........................gr. j,
Sodæ arseniat..........................grs. v,
Hydrarg. bichloridi..........................grs. v,
Potassæ carb, ferri sulph., aa..................ʒ ij.

Flat pil. No. cxx. One pill for a dose three or four times a day after food.

Where a patient has an aversion for pills, as many have, particularly in such a quantity and for such a length of time, I prescribe as a substitute the following mixture :

℞. Hydrarg. bichloridi..........................grs. iss,
Sodæ arseniat..........................grs. iij,
Strychniæ sulph..........................gr. j,
Vini ferri amara..........................f. ℥ xvj.

Take small tablespoonful in water after each meal.—*Medical and Surgical Reporter.*

Prof. Da Costa prescribed for a case of *hysteria*, the acute attack having been cured by the valerianate of zinc, the following:

℞. Liq. arsenii chloridi..........................gtts. v,
Tinct. ferri chloridi..........................gtts. xv,
Syrup. simp..........................gtts. x,
Elixir simp., ad..........................f. ʒ j.

M. Sig. This dose to be taken *ter die*, after meals, well diluted.
Every night this pill to be taken :

℞. Aloin..........................gr. 1-10,

Ex. belladonnæ..........................gr. 1-12,

Rhei..........................gr. ij.

—*Medical World.*

**Scarlet Fever.**—A writer in Medical World says: I think Dr. L. H. Washington's treatment in scarlatina is a very excellent one, and one that is quite generally used. We have considerable scarlatina here in our country this winter, but mostly of the simple form. As a prophylactic, diuretic and febrifuge, I have had a happy result from the following :

℞. Liq. amon. acet..........................℥ j,

Spts. aeth. nit..........................℥ j,

Syr. sim..........................℥ ij,

Sanguinariæ nit..........................gr. i.

M. Sig. Teaspoonful three times a day to a child two to five years old.

